# Towards functional maps of non-coding variants in cancer

**DOI:** 10.3389/fgeed.2024.1481443

**Published:** 2024-10-31

**Authors:** Yihan Wang, Gary C. Hon

**Affiliations:** ^1^ Cecil H. and Ida Green Center for Reproductive Biology Sciences, University of Texas Southwestern Medical Center, Dallas, TX, United States; ^2^ Division of Basic Reproductive Biology Research, Department of Obstetrics and Gynecology, Department of Bioinformatics, University of Texas Southwestern Medical Center, Dallas, TX, United States

**Keywords:** functional genomics, enhancers and promoters, genetic variants, cancer, gene regulation, noncoding regulatory regions

## Abstract

Large scale cancer genomic studies in patients have unveiled millions of non-coding variants. While a handful have been shown to drive cancer development, the vast majority have unknown function. This review describes the challenges of functionally annotating non-coding cancer variants and understanding how they contribute to cancer. We summarize recently developed high-throughput technologies to address these challenges. Finally, we outline future prospects for non-coding cancer genetics to help catalyze personalized cancer therapy.

## The challenges of interpreting non-coding variant function in cancer

Understanding the molecular mechanism of a cancer variant has clear implications to treatment. For example, KRAS is frequently mutated in several cancers and the G12C mutation locks KRAS into an active oncogenic state. Functional profiling of mutant KRAS led to the development of a new class of inhibitors that were recently FDA approved for patients with KRAS G12C mutations (Ostrem et al., 2013; Lito et al., 2016; Janes et al., 2018; Canon et al., 2019). This example stresses the need to 1) map cancer variants and 2) understand their mechanisms. There are different kinds of cancer variants, and they can be grouped based on the technologies used to identify them.

• *Somatic mutation identified by sequencing studies*: Somatic mutations are variants found by comparing the tumor and non-tumor samples from the same patient, including point mutations, copy number variants and structural variants. In this review, we focus on the point mutations category. Most of the somatic mutations are acquired. The Cancer Genome Atlas (TCGA) has mapped many coding somatic mutations in cancer with whole exome sequencing, focusing on protein coding regions of the genome (Weinstein et al., 2013; Bailey et al., 2018; Ding et al., 2018). The KRAS G12C example mentioned above is one example of a coding somatic mutation. Recently, the International Cancer Genome Consortium (ICGC) broadly applied whole genome sequencing (WGS) to identify millions of somatic mutations in non-coding regions, which span >95% of the human genome (ICGC/TCGA Pan-Cancer Analysis of Whole Genomes Consortium, 2020; Rheinbay et al., 2020). In addition, sequencing studies can also identify germline variants with cancer relevance (Huang et al., 2018). Excellent online resources such as COSMIC, the cBio cancer genome portal, and CNCDatabase now catalog these sequenced variants (Cerami et al., 2012; Tate et al., 2019; Liu et al., 2021).
• *Single nucleotide polymorphisms (SNPs) identified by Genome-wide association studies (GWAS):* By comparing the allelic frequency in large populations of patients and controls using genotyping arrays, GWAS have identified many inherited cancer-associated SNPs, including breast cancer, ovarian cancer and prostate cancer (Turnbull et al., 2010; Michailidou et al., 2013; Michailidou et al., 2015; Michailidou et al., 2017; Milne et al., 2017; Zhang et al., 2020). Like somatic mutations, many GWAS SNPs are also enriched in non-coding parts of the genome (Corradin and Scacheri, 2014). Public resources for cancer-relevant GWAS SNPs include the GWAS Catalog and PLCO (Ruan et al., 2022; Sollis et al., 2023).

Somatic variants and GWAS variants have different implications for cancer development. First, GWAS variants are associated with increased cancer risk while somatic variants can drive cancer development. GWAS variants are derived from comparing the blood samples from patient and control populations, which are mostly inherited, but somatic mutations compare the tumor and non-tumor samples from the same patient, reflecting personalized acquired variation. Second, somatic mutations precisely point to single base pair change of function during cancer development while most GWAS variants indicate a risk locus. Although somatic mutations are at single-base resolution, the sample size is smaller than cancer GWAS studies, which makes it difficult to distinguish passenger mutations and driver mutations.

**FIGURE 1:**
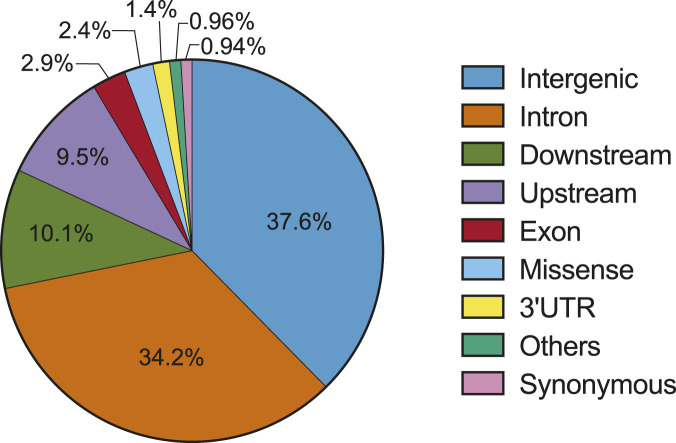
Distribution of somatic variants in the genome (source: PCAWG) (ICGC/TCGA Pan-Cancer Analysis of Whole Genomes Consortium, 2020).

While numerous cancer-associated variants have been identified by different studies (Table 1), elucidating the role of the majority, especially those in non-coding regions, remains a challenge. Unlike coding variants with predictable effects on amino acids, non-coding variants pose unique challenges. First, non-coding variants impact diverse elements in the genome with unique functions. Thus, non-coding variants can influence cancer development through different mechanisms, for example by altering the activity of regulatory elements, modifying gene splicing, and altering miRNA function (described in the next section). Second, the number of non-coding variants far surpasses that of coding variants (Figure 1 (ICGC/TCGA Pan-Cancer Analysis of Whole Genomes Consortium, 2020)). Problematically, many non-coding variants could be passengers rather than drivers. Functionally distinguishing the two possibilities is of utmost importance and will require new approaches for systematic functional analysis. Third, while many non-coding variants influence disease by modifying the expression of cancer genes, the assignment of variants to cancer genes may not be straightforward. For example, non-coding variants often localize to intergenic regions, which complicates efforts to understand how they link to cancer-relevant genes, pathways, and phenotypes. Addressing these challenges to a functional understanding of non-coding variants will be important to personalized medicine in cancer.

**TABLE 1:** Public resources for cancer variants.

Coding somatic mutations	Non-coding somatic mutations	GWAS SNPs
TCGA (Bailey et al., 2018; Ding et al., 2018)	PCAWG (ICGC/TCGA Pan-Cancer Analysis of Whole Genomes Consortium, 2020; Rheinbay et al., 2020; Dentro et al., 2021)	GWAS Catalog (Sollis et al., 2023)
ICGC (Hudson et al., 2010; Zhang et al., 2011; Zhang et al., 2019)	CNCDatabase (Liu et al., 2021)	Prostate, Lung, Colorectal and Ovarian (PLCO) Genetic Atlas project (Ruan et al., 2022)
cBio cancer genome portal (Cerami et al., 2012; Gao et al., 2013)	All of Us Research Program (Ronquillo and Lester, 2022; All of Us Research Program Genomics Investigators, 2024)	
COSMIC (Tate et al., 2019)		

This review describes current challenges to non-coding variant interpretation in cancer and introduces recent technological innovations that will decode the non-coding landscape of cancer genomes.

## Diverse functions of non-coding variants in cancer

Non-coding cancer variants can be found at broad classes of regulatory elements including promoters, enhancers, and microRNAs. This functional diversity presents complicates efforts to understand their impact on cancer development.

### Promoter activity alteration

Gene expression is regulated by promoters, which recruit transcription factors and RNA polymerase to initiate transcription. Promoter variants may alter transcription factor binding sites to impact the rate of transcriptional initiation and/or elongation (Perera et al., 2016). One notable example is the telomerase reverse transcriptase (TERT) promoter, which is a hotspot of mutation in multiple cancer types (Landa et al., 2013; Vinagre et al., 2013; Borah et al., 2015). In melanoma, TERT promoter mutations lead to increased transcription due to the generation of new binding motifs for ETS transcription factors (Horn et al., 2013). The mutant TERT promoter also harbors epigenetic features of activity, including decreased DNA methylation and increased enrichment of the histone modification H3K4me3 (Stern et al., 2017). Another example is the SNP309 variant located in the MDM2 promoter, which increases the affinity of transcription activator SP1 (Bond et al., 2004). Since MDM2 is a negative regulator of tumor suppressor p53, this variant indirectly leads to a lower level of TP53, and is associated with accelerated tumor formation. These examples highlight the impact of promoter variants on cancer development.

### Enhancer activity modification

Non-coding variants are especially enriched at transcriptional enhancers (Corradin and Scacheri, 2014), which are promoter-distal regulatory elements that serve as a platform to bind transcription factors and activate gene expression from a distance (Schoenfelder and Fraser, 2019). Since enhancer activity is exquisitely cell type and tissue type specific (Heintzman et al., 2009; Visel et al., 2009), these regulatory elements drive the diverse expression patterns of different cell types. Genome-wide mapping of enhancers through chromatin profiling have consistently illustrated the strong enrichment of enhancers with GWAS variants across many disease contexts including cancer (Visel et al., 2009; Creyghton et al., 2010; Rada-Iglesias et al., 2011; Pradeepa et al., 2016). Several examples underscore the important roles that enhancers play in cancer development (Mansour et al., 2014; Morton et al., 2019; Huang et al., 2021; Leeman-Neill et al., 2023). For example, a gain-of-function non-coding variant in leukemia creates a new MYB binding site, activating a new enhancer that induces the oncogene TAL1 (Mansour et al., 2014). As another example, the FOXC1 enhancer regulates invasion in triple negative breast cancer cells (Huang et al., 2021). Non-coding mutations convert this enhancer to target a different gene (ZCCHC7), which contributes to cancer development by rewiring protein synthesis (Leeman-Neill et al., 2023). Finally, a more dramatic mechanism of enhancer dysregulation in cancer is enhancer hijacking, where chromosomal rearrangements cause enhancer-mediated activation of oncogenes such as MYC (Xu et al., 2022). In summary, genetic variants can alter enhancer activity or enhancer position, propelling cancer development.

### Transcript splicing alternation

Non-coding variants can also drive cancer through alternative splicing. Abnormal splicing widely occurs in multiple cancer types (Jung et al., 2015; Jayasinghe et al., 2018). For example, mutations of BCL2L1 gene induce apoptotic resistance in breast cancer and prostate cancer through up-regulating the anti-apoptotic form of alternative splicing transcripts (Boise et al., 1993; Bauman et al., 2010; Sveen et al., 2016). Another mechanism is that abnormal splicing leads to intron retention of tumor suppressor genes such as ARID1A, PTEN and TP53, which further inactive the function of those tumor suppressor genes (Jung et al., 2015).

### miRNAs dysfunction

Non-coding variants in miRNAs can also contribute to cancer development. miRNAs fine tune gene expression post-transcriptionally by binding to the 3′UTR of target mRNA with complementary sequence, with impacts on translation inhibition or transcript degradation (Lujambio and Lowe, 2012; Bartel, 2018). Cancer associated variants can alter miRNA seed sequences or miRNA binding sites on the 3′UTRs of target transcripts. Systematic analysis has identified cancer mutations enriched in specific miRNAs, which are strongly correlated to cancer gene programs (Urbanek-Trzeciak et al., 2020). One example is hsa-let-7d*,* which is implicated in breast cancer, ovarian cancer and colorectal cancer (Jiang et al., 2018; Wei et al., 2018; Chen et al., 2019; Urbanek-Trzeciak et al., 2020). hsa-let-7d post-transcriptionally regulates multiple oncogenes and tumor suppressors. In breast cancer, has-let-7d negatively regulates the expression of Jab1, a proliferation pathway regulator. In ovarian cancer, has-let-7d blocks the p53 signaling pathway through HMGA1.

In summary, non-coding mutations affect cancer development through several mechanisms. Linking non-coding variants to cancer genes and pathways is a key step to understanding how they contribute to cancer.

## High throughput approaches to functionally annotate cancer variants

**FIGURE 2:**
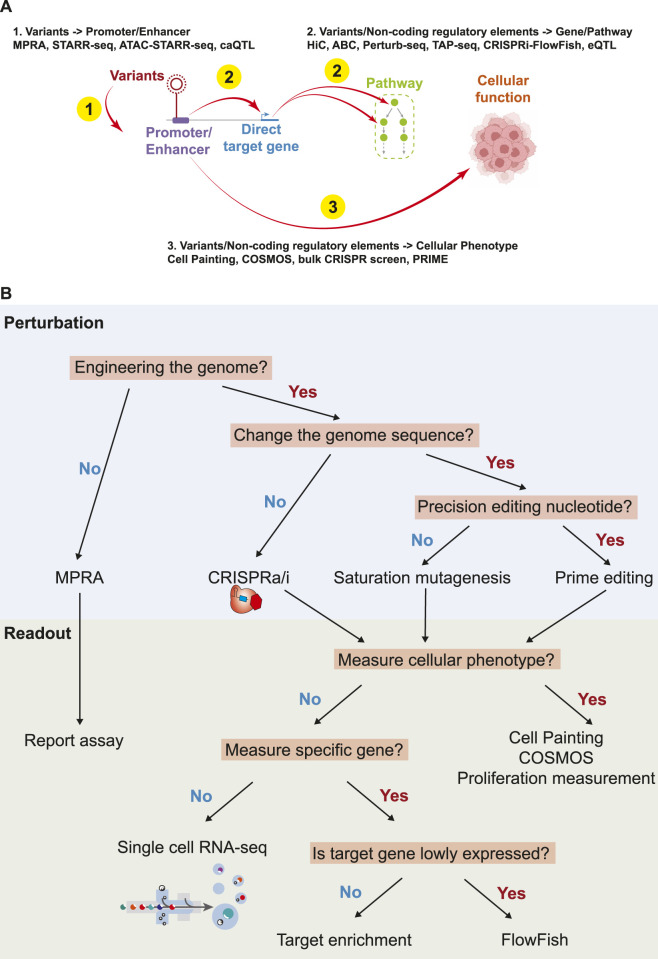
Summary of biological links, and the technologies to understand the links. **(A)**. Summary of technologies to understand the function of non-coding variants/regulatory elements. **(B)**. An overview of perturbations and readouts for high throughput technologies.

The abundance of non-coding mutations in cancer necessitates advanced technologies for comprehensive functional studies (Figure 1). Here, we summarize contemporary technologies for the high-throughput analysis of non-coding variants, with a focus on characterizing the impact of variants and regulatory elements, particularly promoters and enhancers (Figure 2).

### Assessing the impact of non-coding variants on promoter/enhancer activity

Massive Parallel Reporter Assays (MPRA) can simultaneously quantify the activities of millions of promoters and enhancers to drive gene expression (Kircher et al., 2019; Shigaki et al., 2019; Choi et al., 2020; Long et al., 2022). MPRAs are carried out by high throughput cloning of synthetic elements (typically <300 bp) together with transcribed genetic barcodes into a plasmid with a reporter gene, followed by transduction into cells and RNA readout of barcode expression. Importantly, since MPRAs compare the activities of synthesized sequences, they are compatible with the high throughput assessment of non-coding variants compared to control sequences. Although the throughput of MPRAs can be extremely high, one disadvantage is the lack of genomic context. For example, one study applied MPRAs to several hundred melanoma variants and verified multiple variants regulating MX2 activity (Choi et al., 2020). Another study examined more than 1,000 multiple myeloma variants and identified causal variants at six loci (Ajore et al., 2022). Like MPRAs, STARR-seq also quantifies the transcriptional activity of regulatory elements through high throughput reporter assays, with the key difference being that tested sequences are isolated using a biochemical assay like ChIP-Seq or ATAC-Seq (Arnold et al., 2013; Hansen and Hodges, 2022). One study used STARR-seq to systematically identify hundreds of SNPs with the ability to regulate gene expression and to verify that the rs11055880 SNP regulates ATF7IP in breast cancer (Liu et al., 2017). Another recent study applied STARR-seq to find that transposable elements have functional enhancer activity in cancer (Karttunen et al., 2023). The strength of MPRAs and STARR-Seq is the low cost to functionally examine non-coding sequences, which enables large-scale studies of enhancers, promoters, and their variants. However, one key disadvantage is that MRPAs test sequences outside of their native genomic context.

Chromatin accessibility quantitative trait loci (caQTL) studies test the association of genetic variants and chromatin accessibility by performing ATAC-Seq in a large cohort of genetically diverse individuals (Tehranchi et al., 2019; Ajore et al., 2022; Wang et al., 2022). By profiling the chromatin accessibility from cancer patients, one can test if SNPs at promoters and enhancers are associated with gain or loss of function. The approach can be applied with somatic mutation as well. One study in bladder cancer patients identified a somatic variant that generates new binding sites for NKX2-8 with a dramatic increase in open chromatin accessibility, which results in FGD4 upregulation and low patient survival rate (Corces et al., 2018).

### Assessing the impact of variants/non-coding regulatory elements on gene expression

Non-coding variants that alter the activity of promoters and enhancers (previous section) can lead to downstream changes in gene expression and pathway activity to influence cancer development (Bauer et al., 2013). For example, one study documented a non-coding cancer variant that converts an enhancer to target *ZCCHC7*, leading to protein synthesis rewiring and cancer development (Leeman-Neill et al., 2023). In this section, we discuss both computational and experimental approaches to dissect the impact of variants or non-coding regulatory elements to genes and pathways.

While non-coding variants are enriched within enhancers (Corradin and Scacheri, 2014), a key unresolved question is: what are the target genes of these enhancers? Several computational approaches have been developed to address this question. One approach uses three-dimensional chromatin confirmation information, for example with genome-wide HiC data (Lieberman-Aiden et al., 2009), to link enhancers to target genes. One study used HiC to demonstrate that the chromatin structure of the androgen receptor (AR) locus is altered in prostate cancer (Rhie et al., 2019). HiC with single-cell resolution has also been developed to identify cell type specific enhancer regulation (Zhang et al., 2022). One computational approach, the ABC (Activity-by-contact) model, predicts enhancer target genes across the genome as a function of enhancer strength and the 3D chromatin contact frequency (Fulco et al., 2019; Ying et al., 2023). One study demonstrated that an enhancer with variant rs4810856 regulates PREX1, CSE1L and STAU1 expression and activates p-AKT signaling in colorectal cancer (Ying et al., 2023). A recent advance is the development of ENCODE-rE2G, an improved algorithm for predicting enhancer to gene activity with supervised machine learning (Gschwind et al., 2023). However, despite these innovations, current computational approaches are not perfect and are limited by available datasets. As such, the prediction of enhancer targets remains an open problem.

Advances in genome engineering and genomics have catalyzed the development of new approaches to evaluate the functions of non-coding regulatory elements. CRISPR activation (CRISPRa) and repression (CRISPRi) has been frequently employed as a robust tool to modulate the activity of non-coding regulatory elements. One key readout is how these regulatory perturbations impact the expression of target genes and the activity of pathways, by profiling the expression of specific genes, gene subsets, or whole transcriptomes. Perturb-seq combines CRISPRa/i and single cell RNA-seq to measure the impact of non-coding regulatory element perturbation at high throughput (Adamson et al., 2016; Xie et al., 2017; Xie et al., 2019; Gasperini et al., 2019). Perturb-seq has also been applied in cancer cells to facilitate the construction of gene regulatory networks (Dietlein et al., 2022; Ursu et al., 2022; Wang Y. et al., 2023). By measuring whole transcriptomes, Perturb-Seq also enables the exploration of secondary effects from enhancer perturbation. These indirect linkages between disease associated enhancers and disease genes may explain how genetic variants that are far from disease-causing genes can influence complex diseases (Boyle et al., 2017; Wang Y. et al., 2023). For example, we have shown that variants associated enhancers regulate the cell cycle pathway globally in breast cancer (Wang Y. et al., 2023). Another study demonstrates that enhancers within breast cancer risk loci regulate cell proliferation (Tuano et al., 2023).

While Perturb-Seq is a powerful tool, one disadvantage is its high cost. To address this issue, Targeted Perturb-seq (TAP-seq) has been developed to specifically enrich and sequence a subset of genes in Perturb-seq experiments (Schraivogel et al., 2020). This approach reduces cost and increases the sensitivity to detect lowly expressed genes. However, one disadvantage is that loss of transciptome-wide readout precludes unbiased analyses, which could be addressed by expanding the pool of enriched transcripts (Estilo et al., 2009). An even more specific approach is CRISPRi-FlowFish, which perturbs regulatory elements and uses FACS to sensitively measure changes in gene expression (Arrigucci et al., 2017; Fulco et al., 2019). One study identified five non-coding regulatory elements of XBP1 in breast cancer cells, which are the hotspots of breast cancer mutations (Dietlein et al., 2022). The sensitivity of FlowFish is high, which enables the analysis of lowly expressed genes. In addition, this method reduces the cost of sequencing. However, disadvantages include being limited to analyzing a handful of genes at a time and the need to optimize and validate the FISH probes used to detect transcript expression.

Recent advances have enabled a new suite of tools to examine non-coding functions at nucleotide resolution. Computationally, eQTL analysis can infer the impact of variants on genes, and has been widely used in the cancer setting (Li et al., 2013; Nica and Dermitzakis, 2013; Gong et al., 2018). By correlating variant status and nearby gene expression levels across a large cohort of patients, eQTL analysis assigns non-coding variants to the genes likely being misregulated. For example, ESR1, MYC and KLF4 have been linked to three different risk loci in breast cancer with eQTL analysis (Li et al., 2013). Experimentally, traditional reporter assays and higher throughput MPRAs have been widely used to test the impact of non-coding variants on the expression of a reporter gene *in vitro* (Pomerantz et al., 2009; Long et al., 2010; Dietlein et al., 2022). Finally, variant editing methods such as prime editing/base editing combined with target gene measurement provide a means to precisely quantify the effects of genetic variants in an endogenous genomic context (Canver et al., 2015; Dixit et al., 2016; Martyn et al., 2023). These methods directly edit the genome and measure gene expression, offering a more accurate reflection of variant function. By directly knocking-in disease variants, these experiments can give more relevant insights on the impact of variants compared to approaches like CRISPRa or CRISPRi. For example, correcting TERT promoter mutation using base editing can inhibit the cancer phenotype *in vivo* (Li et al., 2020). In parallel, new developments in saturation genome editing are enabling the functional characterization of all possible single-nucleotide genetic variants of cancer genes such as BRCA1 (Findlay et al., 2018).

### Assessing the impact of variants/non-coding regulatory elements on cellular phenotypes

Cancer cells exhibit multiple hallmarks, and studies have used these features as cellular readouts to quantify the impact of variants (Hanahan and Weinberg, 2000; Hanahan and Weinberg, 2011; Hanahan, 2022). One study has shown that cellular morphology correlates with cancer hallmarks such as metastasis, and morphology can be a predictive marker of cancer cell state (Alizadeh et al., 2020; Wu et al., 2020). There are several methods to measure the morphological phenotype such as Cell Painting and COSMOS (Bray et al., 2016; Salek et al., 2022). Both methods can evaluate cellular morphology in a high-throughput and high-content way. For example, high throughput Cell Painting experiments are able to capture variant phenotypes in lung cancer, and are highly correlated with transcriptional phenotypes (Caicedo et al., 2022). Integrating perturbation and morphological measurement could be a powerful tool to understand the variant impact on cellular level (Way et al., 2021; Haghighi et al., 2022).

An alternative method for assessing cellular phenotype involves a focus on biological phenomena, particularly cancer-associated processes such as proliferation, migration, and apoptosis. For example, traditional (bulk) CRISPR screens (Tsherniak et al., 2017). Identify genetic perturbations that either increase or decrease the proliferation of cancer cells. DepMap contains whole genome CRISPR screens across a wide panel of diverse cancer cell lines (Tsherniak et al., 2017). Extending this approach to individual variants, PRIME uses prime editing to install variants into cancer cells and then identify the variants that accelerate proliferation in a cancer context (Ren et al., 2023).

## Perspectives

### The challenges and future prospects of functionally characterizing enhancer variants in cancer

Mapping the variant-gene-pathway-disease network is an active area of current research. But several key challenges remain.

• First: effect size. Numerous studies confirm the limited impact of one single cancer-associated variant (Park et al., 2010; Freedman et al., 2011). The vast majority of variants exhibit low penetrance, contributing to the “missing heritability” problem. This phenomenon extends beyond cancer to complex traits (Purcell et al., 2009; Yang et al., 2010).
• Second: population diversity. Large-scale efforts like whole genome sequencing (WGS) and genome-wide association studies (GWAS) to profile genetic variants have been biased toward certain ancestral populations. Increasing cohort diversity will expand the catalog of variants linked with disease.
• Third: indirect regulation. Many studies have shown that non-coding regulatory regions can regulate genes indirectly (Bauer et al., 2013; Wang Y. et al., 2023). Variant-associated regulatory regions may regulate cancer genes indirectly, through non-obvious mechanisms. New studies using Perturb-Seq and related approaches are needed to comprehensively map these regulatory interactions.
• Fourth: the synergistic variant effects. Multiple non-coding variants can synergistically act on the same gene, complicating variant functional studies. Studies have shown that multiple enhancers can synergistically co-regulate the same target gene (Corradin et al., 2014). Since a single enhancer usually does not fully control a target gene’s expression, it is likely that multiple variants across multiple enhancers are required to alter expression, with each individual variant contributing a mild effect. These attributes add to the difficulty of functionally mapping variant effects. Addressing this challenge requires simultaneous characterization of multiple variants, which is extremely difficult due to the combinatorial complexity of this analysis.
• Fifth: cell type specificity. A tumor is a heterogeneity entity. Variants in non-cancerous cells like fibroblasts and immune cells can also impact therapeutic outcomes by altering the tumor microenvironment (Dhainaut et al., 2022). Thus, it is also important to examine variant functions across cellular contexts. Recognizing the intrinsic heterogeneity of tumors, understanding cell-cell interactions within the tumor microenvironment becomes pivotal. Importantly, current studies have mainly studied variant functions in cancer cell lines and mouse models. Future efforts will need to leverage co-culture or 3D cancer organoid models consisting of multiple cell types, which enhances the likelihood of identifying hits in crucial cancer pathways such as angiogenesis, migration, and immune response (Yuan et al., 2022; Polak et al., 2024).

The omnigenic model posits that a disease or a trait is controlled by a small number of ‘core genes’, and many ‘peripheral genes’ (Boyle et al., 2017; Wray et al., 2018). Core genes directly lead to disease progression, such as tumor suppressors and oncogenes in cancer. Peripheral genes influence core genes, and can include genes like transcriptional regulators. Viewed in this way, variants integrate into the omnigenic model by directly or indirectly influencing core genes or peripheral genes. In this way, the omnigenic model can be readily extended to cancer development. This complex variant-gene regulatory network could possibly explain the small effect size of most cancer variants.

## Concluding remarks

Interpreting non-coding variants remains a significant problem in cancer genetics. Powerful new technologies will facilitate the systematic functional characterization of non-coding variants. However, increases in experimental throughput alone will not be sufficient to understand the function of all cancer variants across all cell states. New computational approaches that learn from these datasets to derive accurate predictions will also be an integral component to a comprehensive understanding of how non-coding variants contribute to cancer (but are outside the scope of this review) (Ostroverkhova et al., 2023; Wang Z. et al., 2023).
